# Editorial: Anemia in children: from nutritional deficits to genetic disorders

**DOI:** 10.3389/fped.2025.1678056

**Published:** 2025-08-22

**Authors:** Sachith Mettananda, Duantida Songdej, Nirmani Yasara

**Affiliations:** ^1^ Department of Paediatrics, University of Kelaniya, Ragama, Sri Lanka; ^2^ University Paediatrics Unit, Colombo North Teaching Hospital, Ragama, Sri Lanka; ^3^ Hematology-Oncology Unit, Department of Pediatrics, Faculty of Medicine Ramathibodi Hospital, Mahidol University, Bangkok, Thailand; ^4^ Department of Biochemistry and Clinical Chemistry, University of Kelaniya, Ragama, Sri Lanka

**Keywords:** anemia, thalassemia, sickle cell anemia, hereditary spherocytosis, Diamond-Blackfan anemia (DBA)

**Editorial on the Research Topic**
Anemia in children: from nutritional deficits to genetic disorders

Anemia is one of the most common health problems among children globally (1). The prevalence of anemia among preschool children ranges between <10% in developed countries in Europe and North America to >50% in developing countries in sub-Saharan Africa (2, 3). The causes for anemia are numerous and include nutritional deficiencies, hemoglobinopathies, inherited red cell disorders, bone marrow failure, and chronic infections (4). The relative contribution of these etiologies to the burden of anemia among children varies from country to country.

This research topic aims to provide insight into many outstanding issues related to anemia in children. The seven manuscripts of this Research Topic examine different aspects related to various causes of anemia among children, ranging from nutritional problems to genetic diseases in different parts of the world.

A study done in Uganda by Komakech et al., published in this research article collection, reported the prevalence of anemia among children aged 6–59 months as 67%. The study highlighted malaria as a significant cofactor, with children who had a history of malaria showing a 20% higher risk of anemia. It also showed that children with older caregivers were more likely to be anemic, while children aged between 3 and 5 years had a lower risk. This emphasizes how local health conditions and family factors influence anemia risk.

The study by Akindutire et al., done in Gambia, uses structural equation modelling to identify sociodemographic determinants of childhood anemia. It identified parental educational attainment, housing location, type of restroom, gender, level of education, marital status, drinking water source, state, number of children, and income status as factors affecting anemia in children.

In addition to the above community-based research studies, the research topic collection published papers covering inherited genetic diseases causing anemia in children. These include sickle cell disease, thalassemia, hereditary spherocytosis, Diamond-Blackfan anemia, and bone marrow failure syndromes.

An observational study done in Senegal by Petigas et al. described how the introduction of neonatal screening impactfully changed the trajectory of sickle cell disease in the country. Infants diagnosed at birth through screening had fewer complications and needed fewer interventions than those who were diagnosed later, when symptoms had already developed. This demonstrates how early diagnosis can reduce suffering and improve long-term health outcomes of children with sickle cell disease.

The thalassemia study reported in this article collection reviews the mechanisms of cardiac injury due to iron overload in thalassemia. Regular blood transfusions are the leading cause of iron overload in children with transfusion-dependent thalassemia (5). The review by Fu and Yang showed that cardiac iron overload affects about a quarter of patients with beta-thalassemia major, contributing to heart failure and early death. The paper describes plausible mechanisms of injury, including oxidative stress and ferroptosis in the heart, and emphasizes the importance of early diagnosis and careful monitoring.

Another brief research report describes the clinical characteristics of a cohort of 64 patients with hereditary spherocytosis in China. The study by Cheng et al. presents the clinical features and correlates the clinical phenotype with genotypes of these patients. The study revealed that patients with genetic variants in *ANK1* and *SPTB* genes have severe disease, whereas *SPTA1* variants are associated with a milder disease.

Diamond-Blackfan anemia (DBA) is another genetic disease that causes anemia through pure red blood cell aplasia. The recent case report by Zhou et al., published under this research topic, described a 56-day-old infant with severe DBA presenting in shock due to an extremely low hemoglobin level of 1.8 g/dl; a rare presentation of the disease. The case report highlights the importance of establishing early genetic testing in patients with DBA before life-threatening complications arise.

The final paper in the collection presents a retrospective analysis of early clinical and laboratory features of 167 primary bone marrow failure syndromes in children in Shandong, China. The study by Leng et al. describes how clinical manifestations, peripheral blood counts, reticulocyte levels, red blood cell indices, and bone marrow examination findings help to differentiate aplastic anemia from refractory cytopenia and idiopathic cytopenia of undetermined significance.

Overall, the research topic has provided insight into the need for multifaceted responses in combating anemia in children. For genetic diseases like thalassemia and sickle cell disease, early diagnosis by genetic testing plays a pivotal role (6). Neonatal screening programs for sickle cell disease, thalassemia, and other inherited conditions can identify affected infants before complications arise. Genetic testing for infants presenting with unexplained severe anemia should be standard practice where resources permit (7).

From a public health viewpoint, improving overall social conditions would have a positive impact on reducing the burden of anemia, especially in underdeveloped countries. Nutrition interventions should be locally designed, including caregiver education on good dietary practices and discouraging harmful habits, like excessive tea consumption with meals (8, 9). Addressing infectious diseases, particularly malaria, remains crucial (10). Without these decisive actions, the combined burden of nutritional and genetic anemia will continue to affect growth, learning, and life opportunities for millions of children around the world.

## References

[1] Collaborators GA. Prevalence, years lived with disability, and trends in anaemia burden by severity and cause, 1990–2021: findings from the global burden of disease study 2021. Lancet Haematol. (2023) 10(9):e713–34. 10.1016/S2352-3026(23)00160-6 37536353 PMC10465717

[2] Tesema GA Worku MG Tessema ZT Teshale AB Alem AZ Yeshaw Y Prevalence and determinants of severity levels of anemia among children aged 6–59 months in sub-Saharan Africa: a multilevel ordinal logistic regression analysis. PLoS One. (2021) 16(4):e0249978. 10.1371/journal.pone.0249978 33891603 PMC8064743

[3] Stevens GA Finucane MM De-Regil LM Paciorek CJ Flaxman SR Branca F Global, regional, and national trends in haemoglobin concentration and prevalence of total and severe anaemia in children and pregnant and non-pregnant women for 1995–2011: a systematic analysis of population-representative data. Lancet Glob Health. (2013) 1(1):e16–25. 10.1016/S2214-109X(13)70001-9 25103581 PMC4547326

[4] Mettananda S de Silva DG. Anaemia in children: are we using the correct prevention strategies? Ceylon Med J. (2017) 62(2):73–6. 10.4038/cmj.v62i2.8469 28697539

[5] Suriapperuma T Peiris R Mettananda C Premawardhena A Mettananda S. Body iron status of children and adolescents with transfusion dependent beta-thalassaemia: trends of serum ferritin and associations of optimal body iron control. BMC Res Notes. (2018) 11(1):547. 10.1186/s13104-018-3634-9 30071883 PMC6071405

[6] Quarmyne M-O Bock F Lakshmanan S Attell BK Snyder A Boudreaux J Newborn screening for sickle cell disease and thalassemia. JAMA Health Forum. (2025) 6(3):e250064. 10.1001/jamahealthforum.2025.0064 40053336

[7] Russo R Marra R Rosato BE Iolascon A Andolfo I. Genetics and genomics approaches for diagnosis and research into hereditary anemias. Front Physiol. (2020) 11:613559. 10.3389/fphys.2020.613559 33414725 PMC7783452

[8] Samararathna R Gunaratne AVC Mettananda S. Knowledge and practices on childhood anaemia, thalassaemia and iron deficiency among mothers of children aged between 6 and 59 months in a suburban area of Sri Lanka. J Health Popul Nutr. (2022) 41(1):59. 10.1186/s41043-022-00341-7 36587235 PMC9805672

[9] Mettananda S Athapathu AS. 1.10 - Iron deficiency anemia. In: Rezaei N, editor. Comprehensive Hematology and Stem Cell Research. 1st ed. Oxford: Elsevier (2024). p. 172–95.

[10] White NJ. Anaemia and malaria. Malar J. (2018) 17(1):371. 10.1186/s12936-018-2509-9 30340592 PMC6194647

